# Factors associated with physical and sexual violence by police among people who inject drugs in Ukraine: implications for retention on opioid agonist therapy

**DOI:** 10.7448/IAS.19.4.20897

**Published:** 2016-07-18

**Authors:** Oksana Kutsa, Ruthanne Marcus, Martha J Bojko, Alexei Zelenev, Alyona Mazhnaya, Sergii Dvoriak, Sergii Filippovych, Frederick L Altice

**Affiliations:** 1 AIDS Program, Section of Infectious Diseases, Yale University School of Medicine New Haven, CT, USA; 2 ICF Alliance for Public Health, Kiev, Ukraine; 3 Ukrainian Institute on Public Health Policy, Kiev, Ukraine; 4 Division of Epidemiology of Microbial Diseases, Yale University School of Public Health New Haven, CT, USA

**Keywords:** police violence, opioid agonist therapy, methadone, buprenorphine, HIV, substance use disorders, Ukraine

## Abstract

**Introduction:**

Ukraine's volatile HIV epidemic, one of the largest in Eastern Europe and Central Asia, remains concentrated in people who inject drugs (PWID). HIV prevalence is high (21.3% to 41.8%) among the estimated 310,000 PWID. Opioid agonist therapy (OAT) is the most cost-effective HIV prevention strategy there, yet OAT services are hampered by negative attitudes and frequent harassment of OAT clients and site personnel by law enforcement. This paper examines the various types of police violence that Ukrainian PWID experience and factors associated with the different types of violence, as well as the possible implications of police harassment on OAT retention.

**Methods:**

In 2014 to 2015, we conducted a cross-sectional survey in five Ukrainian cities with 1613 PWID currently, previously and never on OAT, using a combination of respondent-driven sampling, as well as random sampling. We analysed correlates of police violence by multiple factors, including by gender, and their effects on duration of OAT retention. Self-reported physical and sexual violence by police were the two primary outcomes, while retention on OAT was used as a secondary outcome.

**Results:**

Overall, 1033 (64.0%) PWID reported being physically assaulted by police, which was positively correlated with currently or previously being on OAT (69.1% vs. 60.2%; *p*<0.01). HIV prevalence rates were higher in those receiving OAT than those not on OAT (47.6% vs. 36.1%; *p*<0.01). Police violence experiences differed by sex, with men experiencing significantly more physical violence, while women experienced more sexual violence (65.9% vs. 42.6%; *p*<0.01). For PWID who had successfully accessed OAT, longer OAT retention was significantly correlated both with sexual assault by police and fewer non-fatal overdoses.

**Conclusions:**

Police violence is a frequent experience among PWID in Ukraine, particularly for those accessing OAT, an evidence-based primary and secondary HIV prevention strategy. Police violence experiences, however, were different for men and women, and interventions with police that address these sexual differences and focus on non-violent interactions with PWID to improve access and retention on OAT are crucial for improving HIV prevention and treatment goals for Ukraine.

## Introduction

Ukraine, with an HIV prevalence of 1.2% among adults aged 15 to 49, faces one of the largest and most volatile HIV epidemics in Eastern Europe and Central Asia [1]. With a population of 45.5 million people, Ukraine's HIV epidemic is still predominantly concentrated in people who inject drugs (PWID), with an exceedingly high HIV prevalence (21.3% and 41.8%) among the country's estimated 310,000 PWID [2,3]. Opioid agonist therapy (OAT) with buprenorphine was first introduced in Ukraine in 2004 [4,5], followed by methadone maintenance treatment in 2008 [6]. OAT is one of the most effective and cost-effective strategies for treatment of opioid addiction and prevention of primary and secondary HIV infections for PWID in Ukraine [7–9]. OAT has a number of direct treatment benefits, including reduction in drug injection risk-taking [10] and improvements in quality of life for PWID through improved social functioning, employment, underlying psychiatric disorders and reducing criminal behaviour [11–14].

Despite the documented effectiveness of OAT, scale-up in Ukraine has been hindered by numerous barriers, including police harassment, human rights infringements, ineffective policies, myths about OAT and hostile attitudes toward those who attempt to enter OAT programmes [15–19]. For example, to initiate OAT, PWID are required to officially “register” as an “addict” at government-sponsored narcology (a Soviet term used for the treatment of addiction) clinics. This requirement often deters patients from seeking treatment due to fear of losing certain societal privileges such as employment and driver's license; it potentially allows police to know patients’ identity [15,16,20]. Police have been known to monitor and visit narcology clinics in Ukraine in attempts to obtain information about drug users, demand money from them or extract information about other drug users [21].

The prevailing view by Ukrainian law enforcement, who often interpret and impose harsh regulations toward drug users and utilize harassment to obstruct OAT access, is that drug dependence is more a criminal act rather than a chronic and relapsing medical condition that can be effectively treated [19]. Consequently, police rely on punitive rather than rehabilitative measures to address opioid dependence [20], which undermines HIV prevention and treatment efforts. We therefore hypothesized that police hostility and harassment may negatively influence OAT entry and retention through several mechanisms. First, PWID may not enter or remain on OAT if they have been physically abused by police or know of others who have experienced physical or sexual abuse. Second, police may harass PWID through unofficial detentions for up to three days, long enough to disrupt OAT or antiretroviral medications and precipitate opioid withdrawal symptoms or promote development of antiretroviral medication resistance, respectively [18]. Third, police harass patients at OAT and needle-exchange sites by conducting searches and confiscating HIV medications due to allegedly mistaking them for illegal drugs [16]. Fourth, physical abuse by police officers has also been reported by PWID [22].

This study examines the various types of police violence and factors associated with police violence experienced by Ukrainian PWID, as well as the possible implications of police harassment on retention in OAT.

## Methods

From January 2014 to March 2015, a self-administered survey was conducted in five Ukrainian cities among three groups of PWID: 1) those currently on OAT; 2) those previously on OAT; and 3) those never on OAT. All survey respondents were PWID over 18 years old, who met DSM-V criteria for opioid dependence using a validated, self-report screening instrument [23].

### Participant recruitment

Study participants who were currently or previously on OAT were randomly selected from client lists obtained from OAT sites in each city, while those who were never on OAT were recruited using respondent-driven sampling (RDS). Using standardized methods [24,25], RDS seeds were selected based on residential location, age (±25 years), injection duration (±2 years) and gender. Each RDS participant received three coupons to recruit others. All participants underwent HIV counselling and two-step rapid HIV testing, followed by a standardized survey using a computer-assisted survey instrument (CASI). Participants were paid 100 UAH (approximately $4 to $10 US during the data collection period) for completing the interviews, as well as 20 UAH (approximately $1 to $2 US) for up to three participants they recruited into the study [26]. Research assistants described the research study and obtained written consent. Participation was voluntary and participants could discontinue the survey for any reason and at any time without affecting the benefits or services they were receiving. Consenting participants were assigned an anonymous unique identifier. The study received approval from institutional review boards at Yale University, the Ukrainian Institute for Public Health Policy and the Gromashevskiy Institute at the National Academy of Medical Sciences.

### Study definitions

In addition to socio-demographic characteristics, relationship status was defined as stable if legally married or unmarried but cohabitating. Drug use characteristics included number of years injecting and frequency of injection in the past 30 days, length of time on OAT, frequency of incarceration and reason for last incarceration (related to substance use or a violent crime), and drug dealing as a source of income.

The primary study outcomes consisted of two standardized indicators measuring lifetime 1) physical and 2) sexual violence enacted by police. Physical violence consisted of self-reported experience of physical assault by police officers, including slapping, kicking, punching, slashing, strangling or choking, whereas sexual violence consisted of self-reported non-consensual, forced sexual contact, including oral, vaginal and anal, as well as sex with multiple partners. In addition to physical and sexual violence, we assessed other types of police harassment toward PWID including verbal and physical threats, monetary exhortations, unprovoked detention, particularly on the way to or from an OAT site, and blackmail (including forced cooperation in exchange for avoiding an arrest).

The secondary outcome, retention in OAT, was defined only in current and previous OAT groups as a continuous variable of the number of months that the respondents participated in the methadone or buprenorphine programme.

### Statistical analysis

First, we compared the various characteristics of participants who had received OAT (either currently or previously) and those who had never been on OAT. Chi-square tests were used to compare the frequencies in categorical variables, including gender, relationship status, HIV status and all police violence variables, as well as the methadone and buprenorphine entry and retention variables. A *t*-test was used for all continuous variables. Second, we compared the prevalence of different types of police harassment between two gender groups using chi-square tests with application of sampling weights based on population estimates in each OAT strata by city. The population estimates for the OAT groups were taken directly from the administrative records of OAT programmes, as well as being based on medical records, while the population size of those never on OAT was taken from the previously available estimates and adjusted based on a sample selection of opioid injectors [2]. Third, weighted multivariate logistic regressions were used to analyse the primary outcomes: police physical and sexual violence. Explanatory variables were included in the final estimation based on bivariate associations with *p*<0.10 and whether they had a plausible association with outcomes of interest or whether the variables served as important controls to counter omitted variable bias. Fourth, using retention on OAT as a secondary outcome, we estimated Cox proportional hazard models. The outcome was censored for individuals who were on OAT at the time of the survey. As in the case of logistic regression, the final model incorporated variables that were deemed plausible controls to counter omitted variable bias and produced relative goodness-of-fit based on an Akaike information criterion. The entire set of variables from which a smaller subset of covariates was chosen included demographic characteristics, self-reported non-fatal overdose in the past 30 days, frequency and duration of injection, incarceration history, types of offense for which the person was most recently incarcerated, income from illegal activities, lifetime sexual and physical violence, police harassment and city of residence. Statistical analyses were performed using STATA v 14 [27].

## Results

### Socio-demographic characteristics

Table 1 describes the descriptive characteristics of the 1613 PWID, stratified by those who were currently and previously on OAT (*n=*702), and those never on OAT (*n=*911). Most participants were male (*n*=1233) and in their mid-30s (mean=35.2 years; SD=0.26). OAT participants were significantly older than those never on OAT (38.1 years vs. 35.0 years; *p*<0.01), had injected longer (19.6 vs. 15.3 years; *p*<0.01), reported fewer days of injection in the past month (5.2 vs. 21.4; *p*<0.01) and were more often in stable relationships (39% vs. 32%; *p*<0.01). OAT participants also experienced more physical assault by police (69.1% vs. 60.2%; *p*<0.01) and higher rates of HIV infection (47.6% vs. 36.1%; *p*<0.01). Participants who had never been in OAT reported drug dealing as an additional source of income (5.9% vs. 3.2%; *p*=0.02). The mean duration of OAT treatment for current or previous OAT participants was 9.6 months (SD=1.8). Most participants reported that their most recent incarceration was related to a violent crime (66.9%) rather than a substance use-related one (25.4%).

**Table 1:** Comparison of demographic and risk behaviours among PWID in five cities in Ukraine

	Total sample^a^	Currently or previously on OAT	Never on OAT	
				
Variables	*n* (%)	*n* (%)	*n* (%)	*p*
	*N*=1613	*n*=702	*n=*911	
Mean age, years (SE)	35.2 (0.26)	38.1 (0.36)	35.0 (0.27)	<0.01
Gender				
Male	1233 (76.4)	533 (75.9)	700 (76.8)	Referent
Female	380 (23.6)	169 (24.1)	211 (23.2)	0.668
Relationship status				
In a stable relationship	566 (35.1)	274 (39.0)	292 (32.1)	<0.01
Not in a stable relationship	1047 (64.9)	428 (61.0)	615 (67.5)	Referent
Injection characteristics				
Years of injection – mean (SE)	15.6 (0.29)	19.6 (0.38)	15.3 (0.31)	<0.01
Number of days injected any substance (past 30 days) – mean (SE)	20.8 (0.31)	5.2 (4.40)	21.4 (0.32)	<0.01
Months in OAT programme – mean (SE)	1.70 (0.52)	9.6 (1.8)	–	na
Number of incarcerations – mean (SE)	1.09 (0.06)	1.04 (0.08)	1.09 (0.07)	0.91
Last incarceration related to substance abuse	393 (25.4)	183 (26.1)	210 (23.1)	0.16
Last incarceration related to a violent crime	1079 (66.9)	519 (73.9)	615 (67.5)	0.55
Lifetime physical assault by police	1033 (64.0)	485 (69.1)	548 (60.2)	<0.01
Lifetime sexual assault by police	70 (4.3)	32 (4.6)	38 (4.2)	0.71
Drug dealing as an additional income source	77 (4.8)	23 (3.2)	54 (5.9)	0.02
HIV test results				
Positive	663 (41.1)	334 (47.6)	329 (36.1)	<0.01
Negative	950 (58.9)	368 (52.4)	582 (63.9)	Referent

a Averages weighted by population fractions in each OAT strata and city.

OAT, opioid agonist therapy; PWID, people who inject drugs; SE: standard error; NA: not applicable.

### Police harassment and violence

The prevalence of police harassment is shown in Table 2. It differed by gender, with men being more frequently threatened with police violence, physically assaulted by police and physically assaulted with a weapon. Conversely, women reported they were forced by police officers to have sex more often than men (13.1% vs. 1.4%; *p*<0.01). Overall, 22% of OAT participants were stopped by police on the way to or from OAT, with men reporting being stopped more often than women (26.0% vs. 8.9%; *p*<0.01). Men were also significantly more likely than women to be victims of police harassment during required daily OAT attendance, including police officers demanding money as bribes (10.6% vs. 3.2%; *p*<0.01), police officers demanding cooperation with reporting illegal activities by other PWID to avoid arrest (10.0% vs. 3.7%; *p*<0.01) and unofficial police detention that did not result in official charges (10.1% vs. 2.1%; *p*<0.01). Table 3 also demonstrates the prevalence of police harassment in five Ukrainian cities. Dnepropetrovsk had the highest prevalence of both lifetime physical and sexual violence.

**Table 2:** Prevalence of police harassment by gender in five Ukrainian cities

	Total	Men	Women	
				
	*N=*1613	*n=*1233	*n=*380	
				
Variable	*n* (%)	*n* (%)	*n* (%)	*p*
Threatened by police with physical violence (lifetime)	753 (46.8)	626 (50.8)	127 (33.4)	<0.01
Threatened by police with a weapon (lifetime)	534 (33.2)	452 (36.7)	82 (22.0)	<0.01
Physically assaulted by police (lifetime)	971 (60.2)	813 (65.9)	158 (42.6)	<0.01
Physically assaulted by police (last six months)	196 (12.2)	170 (13.8)	26 (6.8)	<0.01
Forced by police to have sex (lifetime)	62 (3.8)	12 (1.4)	50 (13.1)	<0.01
Forced by police to have sex (past six months)	12 (0.7)	4 (0.3)	8 (2.1)	<0.01
Not interested in OAT treatment due to police harassment	396 (24.6)	303 (24.6)	93 (24.5)	0.98
Stopped by police while going to/from OAT (OAT participants only)	355 (22.0)	321 (26.0)	34 (8.9)	<0.01
Police demanded money when stopping to/from OAT	143 (8.9)	131 (10.6)	12 (3.2)	<0.01
Police asked to cooperate when stopping to/from OAT	137 (8.5)	123 (10.0)	14 (3.7)	<0.01
Police detained or arrested when stopped to/from OAT	132 (8.2)	124 (10.1)	8 (2.1)	<0.01

Note that statistics were weighted by the population fractions in each OAT strata and city.

OAT, opioid agonist therapy.

**Table 3:** Prevalence of police harassment by city in Ukraine

	Kiev	Odessa	Mykolayiv	Dnepropetrovsk	Lviv
					
	*N*=413	*N*=315	*N*=344	*N*=380	*N*=273
					
Variable	*n* (%)	*n* (%)	*n* (%)	*n* (%)	*n* (%)
Threatened by police with physical violence (lifetime)	198 (47.9)	115 (36.5)	151 (43.8)	177 (48.0)	112 (41.0)
Threated by police with a weapon (lifetime)	146 (35.3)	90 (28.5)	114 (33.1)	114 (30.9)	70 (25.6)
Physically assaulted by police (lifetime)	255 (61.7)	133 (42.2)	203 (59.0)	238 (64.6)	142 (52.0)
Physically assaulted by police (last six months)	47 (11.3)	20 (6.34)	34 (9.9)	62 (16.8)	33 (12.0)
Forced by police to have sex (lifetime)	14 (3.38)	8 (2.53)	9 (2.61)	23 (6.25)	8 (2.93)
Forced by police to have sex (past six months)	1 (0.24)	1 (0.32)	3 (0.87)	6 (1.63)	1 (0.366)
Not interested in OAT treatment due to police harassment	87 (21.0)	62 (19.6)	89 (25.8)	85 (23.0)	73 (26.7)

Note that statistics were weighted by the population fractions in each OAT strata and city.

OAT, opioid agonist therapy.

Table 4 presents the multivariate analyses of the correlates of physical and sexual violence by police experienced by PWID participants. Lifetime physical violence was positively correlated with being male, longer drug injection duration and the number of incarcerations. Compared to other reasons for incarceration, incarceration related to substance use was negatively correlated with physical violence and not significantly correlated with lifetime sexual assault. Lifetime sexual assault was associated with being female; women were over 10-fold more likely to have experienced sexual violence by police than men (OR=10.73; *p*<0.01). Other correlates of sexual violence by police included years of injection and number of incarcerations; however, reason for incarcerations was not significantly associated with police-related sexual violence.

**Table 4:** Factors associated with physical and sexual assault of PWID by police in Ukraine (*N=*1613)

	Lifetime physical assault by police^a^			Lifetime sexual assault by police^a^		
		
Covariates	OR	95% CI	*p*	OR	95% CI	*p*
Gender	0.37	[0.26, 0.51]	<0.01	10.73	[4.9, 23.1]	<0.01
Years of injection	1.03	[1.01, 1.04]	<0.01	0.99	[0.95, 1.02]	0.498
Number of incarcerations	1.14	[1.01, 1.34]	0.05	1.09	[0.92, 1.28]	0.289
Last incarceration – violent crime	1.19	[0.65, 2.11]	0.57	Referent		
Last incarceration – substance abuse	0.43	[0.25, 0.74]	<0.01	1.13	[0.45, 2.73]	0.791
Last incarceration – other	Referent		–	Referent		–
Income from drug dealing	1.65	[0.86, 3.14]	0.13	2.08	[0.81, 5.27]	0.123
HIV status	–	–	–	1.48	[0.77, 2.82]	0.23
Constant	1.95	[1.1, 3.43]	0.02	0.01	[0.004, 0.03]	<0.01

a Statistics weighted by the population fractions in each OAT strata and city.

OAT, opioid agonist therapy; PWID, people who inject drugs.

### Impact of police violence on retention on OAT

Table 5 highlights the factors correlated with retention on OAT for the 702 participants currently or previously on OAT. PWID who had experienced physical violence by the police had higher, but not statistically significant, relative hazard rates (HR: 1.29, *p*=0.08) for discontinuing OAT and therefore had lower retention on OAT relative to PWID who had never experienced police physical violence. Additionally, experience of police-related sexual violence and overdose was associated with lower relative hazard rates and therefore correlated with longer retention on OAT.

**Table 5:** Factors associated with duration of OAT participation (*N*=702)

Covariates	Hazard ratio for discontinuing OAT	95% CI	*p*
Years of injection	1.00	[0.97, 1.01]	0.68
Experienced nonfatal overdose	0.93	[0.87, 0.99]	0.03
Gender	1.07	[0.80, 1.42]	0.63
Lifetime physical assault by police	1.29	[0.97, 1.72]	0.08
Lifetime sexual assault by police	0.51	[0.28, 0.90]	0.02
Stopped by police going to/from OAT site	1.18	[0.86, 1.62]	0.30

Note that statistics were weighted by the population fractions in each OAT strata and city.

OAT: opioid agonist therapy.

## Discussion

In the largest survey of PWID in Ukraine, experiences with police physical and sexual violence were high for PWID who had accessed OAT as well as those who had never been on OAT. The high prevalence of police harassment on the way to or from OAT sites is especially concerning because scale-up of OAT is the most cost-effective strategy to prevent HIV in Ukraine [9]. Moreover, experiences with police victimization, especially when accessing OAT clinics, are likely to negatively impact on OAT entry and retention and undermine HIV prevention efforts. OAT has been available in Ukraine for over a decade; however, expansion efforts have stagnated for the past five years despite ample funding to expand treatment slots. A number of personal and structural factors such as negative perceptions about OAT medications, especially methadone, strict policies on dosage, inability to take home medications, inconvenient clinic locations and subpar treatment settings have hampered treatment expansion [15,16,28], but data here and elsewhere support that police also negatively influence OAT expansion [18,20,22,29–32].

Although experience of police-related violence is common among PWID, women experience it differently than men, with higher levels of sexual violence. Previous studies from Russia and other countries where PWID are common have reported that females are often sexually abused and raped by police officers [33]. A study of female sex workers (FSWs) in two Russian cities, half of whom reported some history of drug injection, showed that more than one-third of the study participants reported sexual coercion by police [34]. Other studies have found high levels of police sexual misconduct with female drug court attendees, who report trading sex for favours with police officers [35]. Police often search women drug users and confiscate medications, sterile injection equipment and condoms, which places women at greater risk for HIV infection after release by police through needle-sharing and unprotected sex [36]. Women who have been sexually assaulted by police are also at increased risk for psychiatric conditions, such as post-traumatic stress disorder, which may increase HIV risk-taking and influence women to avoid interactions with healthcare delivery programmes or to seek care for conditions not related to victimization [36]. When they do access OAT, however, they have the option to seek additional help from social workers, psychologists or reproductive healthcare workers. Although our study compared males with females, studies of FSWs elsewhere indicate that they experience a substantial burden of police brutality [37]. Sexual violence against FSWs is associated with increased risk of HIV and other sexually transmitted infections (STIs) [37,38]. Although evidence from the general population who do not engage in sex work suggests that violence experienced by women, whether physical or sexual, is not directly correlated with HIV, it increases the risk of exposure to such practices as unprotected sex and unsafe injections, which does increase female vulnerability to HIV and STIs [37–39]. It also contributes to acute and longstanding psychological distress [40,41]. FSWs who report experiences with violence, whether sexual or physical, often report inconsistent condom use, condom failure and coercion into unprotected sex [36,37]. FSWs who also inject drugs may experience needle confiscation by police and therefore practice needle sharing, which leads to increased HIV risk and STIs [36,37]. A specific focus by police to encourage OAT entry is therefore in the best interest of female PWID not only for primary and secondary HIV prevention, but to facilitate treatment for other medical and psychiatric co-morbidities [8].

Although it is not surprising that longer duration of drug injection was associated with more frequent experiences with physical police violence, presumably due to longer periods of being “at risk,” the finding that OAT participants experienced higher levels of police violence after controlling for other factors is concerning. Longer drug injection duration might also explain the higher HIV prevalence among OAT participants. With longer durations of drug injection, OAT participants were exposed to greater HIV risk through participation in high-risk behaviours such as needle-sharing. Therefore, higher HIV prevalence among OAT participants, along with increased physical violence associated with longer duration of drug injection, makes people living with HIV especially vulnerable to physical police brutality. Although we cannot disentangle whether the increased interaction with police occurred specifically as a result of OAT enrolment, our finding of high levels of police harassment while patients travel to and from OAT sites is suggestive of this relationship of police disrupting OAT services. Because OAT in Ukraine requires daily observation of medication-taking, PWID on OAT are especially easy targets for the police. Previous findings from Ukraine also suggest that police target PWID at OAT sites, resulting in confiscation of medications and subjecting them to beatings [18,20,22]. The far-reaching presence of police at OAT sites destabilizes the treatment process and instils anxiety over possible police harassment [32]. The fear of police violence at OAT sites coerces patients into putting their treatment needs aside and prioritizing avoiding police and potential beatings over seeking treatment, consequently decreasing access to and retention in OAT [32]. Because men face frequent physical abuse at the hands of police, male PWID might be more reluctant to enter or remain enrolled in OAT due to the fear of constant police surveillance and harassment [20]. Therefore, when facing the possibility of police encounters, men are at risk of being beaten, coerced, verbally assaulted and arrested [42].

The stigma that surrounds drug use in Eastern European and Central Asian countries encourages law enforcement practices that routinely target PWID [33,42,43]. However, change is crucial for HIV prevention and treatment efforts in a region where one of the most volatile HIV epidemics persists, concentrated among PWID. One option for the police that has been effective elsewhere, rather than targeting PWID for arrest, would be to align their practices with public health, guiding PWID toward treatment with methadone or buprenorphine and helping them avoid incarceration and HIV risk. Alternatively, police could encourage PWID to more effectively access harm reduction services such as needle/syringe programmes [44–46]. In order to promote the expansion of OAT, the Ukrainian government should provide opportunities for law enforcement officers to learn about the many benefits of OAT, including reduced criminal activity. Such programmes should be geared toward educating law enforcement about drug dependence, dismantling myths that surround OAT and supporting police officers to encourage use of harm reduction services. A successful educational programme was recently initiated in Kyrgyzstan focusing on HIV prevention science, policy and occupational safety. Studies indicate that law enforcement officers who received this training were more likely to support PWID by referring them to harm reduction organizations and refraining from confiscation of needles and syringes [47]. By creating a safer environment that promotes public health rather than interdiction, advocating for OAT and needle/syringe programmes is likely to have an influence on reducing police violence. This is accomplished through establishing dialogue between law enforcement, PWID and providers that promote empowerment of PWID to utilize all available resources for harm reduction and addiction treatment. Support for law enforcement would also help to dismantle the myths and stigma that surround drug use and OAT in Ukraine and other Eastern European countries. In the absence of direct intervention with police, alternative strategies to expand OAT delivery to pharmacies and primary care sites would reduce targeting by police by allowing PWID to receive treatment in non-traditional OAT delivery programmes that do not focus on PWID [48].

Findings that show that police violence is associated with lower OAT retention are intuitive. Not surprisingly, among OAT clients, longer retention in treatment was associated with lower likelihood of non-fatal overdose, which has been described elsewhere [49]. Importantly, however, longer OAT retention was associated with nearly half as much sexual violence. One explanation is that markedly reducing drug use while receiving OAT put PWID at increased risk from police harassment (e.g., bribes), but decreased risk from more violent forms of police interactions when PWID attempted to avoid the police because they had indeed been engaging in criminal activity.

Last, brutal law enforcement practices may indirectly discourage PWID from engaging in OAT. Concerns about law enforcement raids on OAT treatment sites have been reported with attempts to confiscate patient records and other private information to potentially target PWID [32]. Consequently, many hospitals refuse to establish OAT treatment programmes due to the frequent reports of police investigations and harassment of clinicians [15,20]. Beyond targeting patients, police officers have threatened doctors in attempts to dissuade them from providing OAT or to coerce doctors to provide care only to certain patients [32]. As a result, lack of trust between patients and doctors may develop if patients perceive that their clinicians are in collusion with the police [6,32].

## Limitations

Although this is the largest cross-sectional survey of PWID in Ukraine, the findings presented here are not without limitations. The cross-sectional nature of this study may only demonstrate associations and not causality [50]. In addition, self-reported information is prone to bias because reports of victimization were not verified. However, CASI was used because this survey technique reduces under-reporting of sensitive information. Moreover, while our random selection from OAT records and RDS recruitment methods seek to recruit representative samples, both strategies may introduce selection bias. Although we did use standardized measures of physical and sexual violence, the measure related to police was self-reported and possibly imprecise. Therefore, it is difficult to establish external validity of the police violence variable by referencing other studies. Notwithstanding these limitations, this study's comprehensive nature allowed the detection of significant findings on the role of police violence on OAT retention.

## Conclusions

Law enforcement violence remains a major barrier to OAT expansion efforts in Ukraine. Physical violence by police is associated with decreased OAT retention overall, especially in male PWID. Ukraine has an urgent need to realign its justice and public health mandates in order to reduce unnecessary and hazardous detentions, searches and confiscations. Minimally, Ukraine should introduce interventions geared towards educating the nation's law enforcement agencies about drug addiction as a medical disease, HIV and the societal benefits of encouraging addiction treatment with OAT and harm reduction engagement. Whereas integration of services at OAT sites has greatly improved health indicators for HIV-infected PWID in Ukraine [51], further programmes that focus on the needs of female PWID are urgently needed to address the prior psychological consequences of sexual violence by police and to reduce future negative consequences. Links should be established between police, OAT clinics, healthcare providers and PWID in order to facilitate the entry of PWID into and retention on OAT. Most importantly, Ukraine's societal perception about the treatment of underlying substance use disorders needs to be realigned and addressed through promoting evidence-based treatment rather than through criminal sanctions.
